# Supplementary material for: Solvent-driven electron trapping and mass transport in reduced graphites to access perfect graphene
Source: Nat Commun. 2016 Aug 10;7:12411. doi: 10.1038/ncomms12411 (PMC4987516; doi:10.1038/ncomms12411)
Supplement: Supplementary Information — Supplementary Figures 1-6, Supplementary Table 1, Supplementary Methods and Supplementary References [file ncomms12411-s1.pdf]

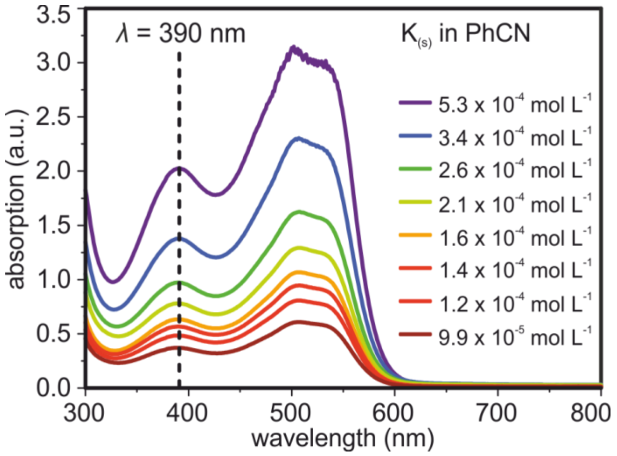

Supplementary Figure 1: UV/Vis absorption spectra of dilution series of pure potassium in benzonitrile. For the quantitative reaction of K + PhCN → K^{+} + $PhCN^{\cdot -}$ the radical anion concentration can be determined by optical spectroscopy under inert conditions. The extinction coefficient: ε_{390} = 4,000 (± 50) L·mol^{-1}·cm^{-1}.

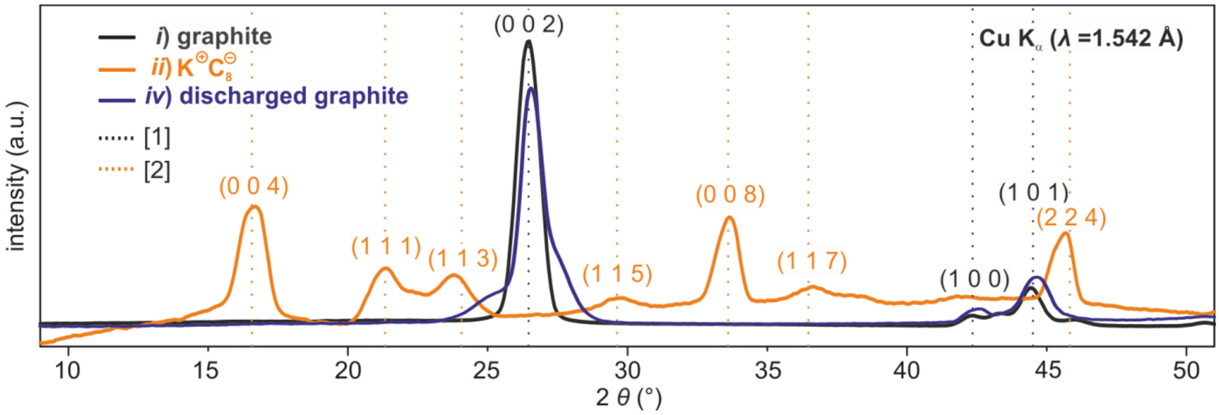

Supplementary Figure 2: XRD patterns of charged and discharged graphite: *i*) pristine graphite, *ii*) intercalated graphite KC_8 and *iv*) KC_8 after discharging by PhCN treatment. The black and orange dashed lines correspond to the of reflections of graphite and KC_8 (full orthorhombic model) reported in literature[1,2], respectively.

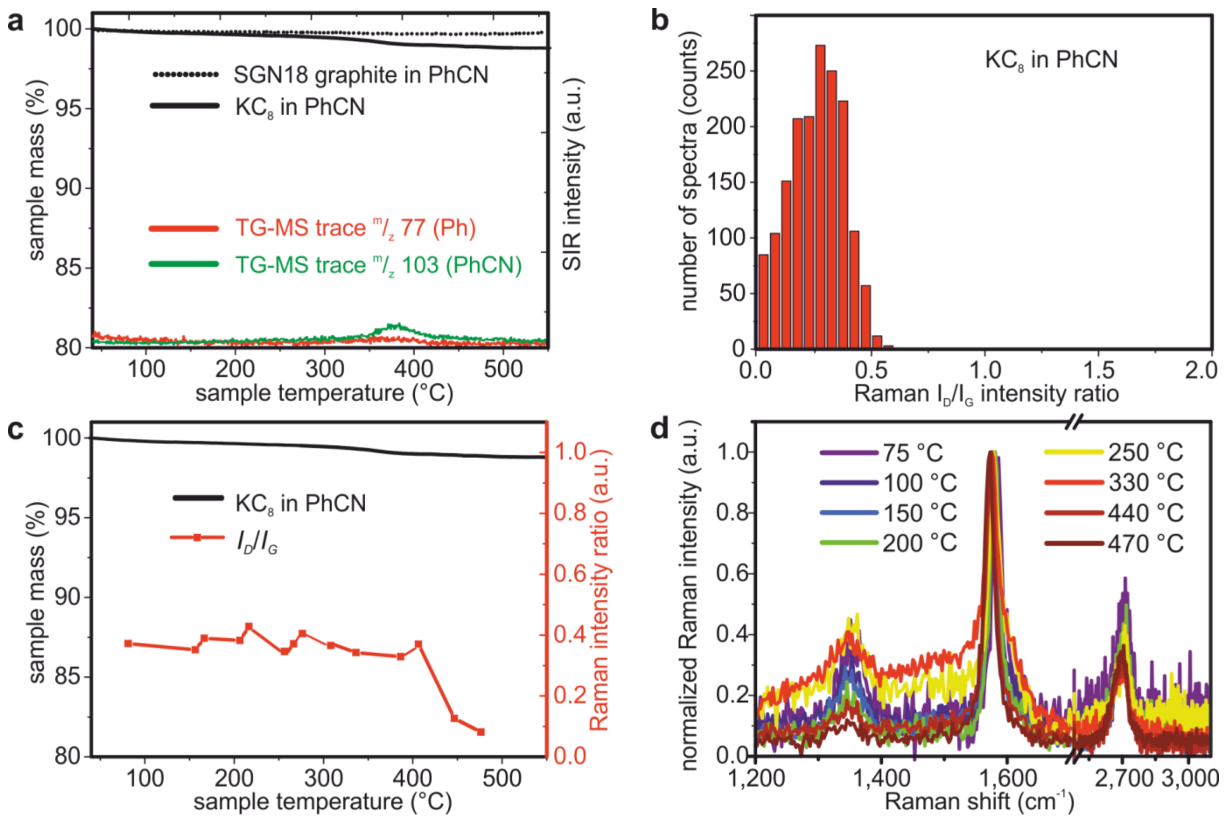

Supplementary Figure 3: Detailed analysis of the graphenide solution of KC_8 in benzonitrile after workup by heating under nitrogen gas atmosphere. a) TG-MS analysis of KC_8 and the reference graphite in benzonitrile. The MS traces for the functional moieties for the phenyl group (m/z 77) and PhCN (m/z 103) show no detection of any fragment being detached from the sample. b) SRS histogram for the Raman I_D/I_G intensity ratios. The distribution matches the histogram of pristine graphite in the Supplementary Figure 6. c) Raman intensity ratios in dependence of the sample temperature measured under inert gas during annealing up to 500 °C. The TG curve is given to demonstrate the correlation; the individual spectra are plotted in d).

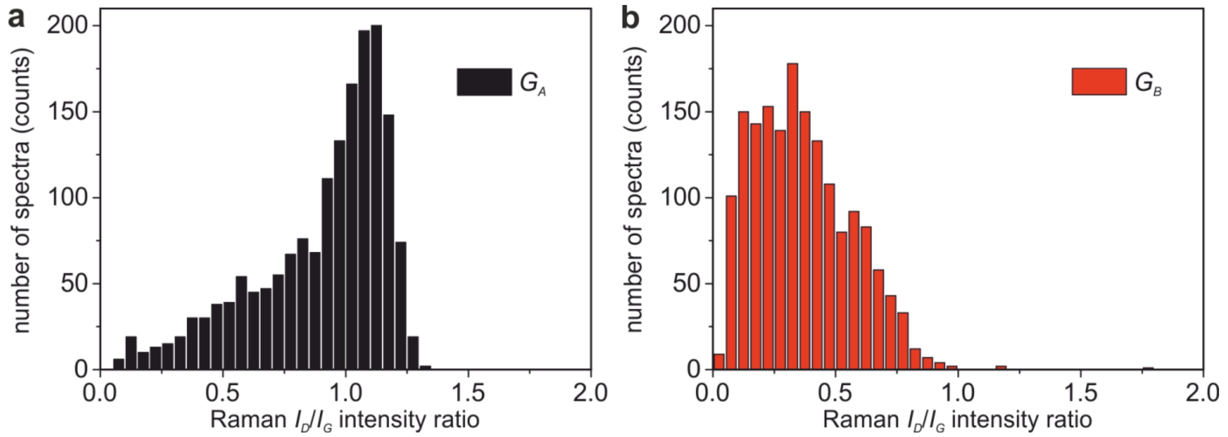

Supplementary Figure 4: SRS analysis showing the Raman I_D/I_G intensity ratio histograms for the graphenide solutions of KC_8 in THF in Figure 3. a) G_A was received by direct exposure to ambient conditions. b) G_B was treated with benzonitrile prior to workup.

Note the already present D-Mode in the starting material reference (G_P) as shown in Supplementary Figure 6.

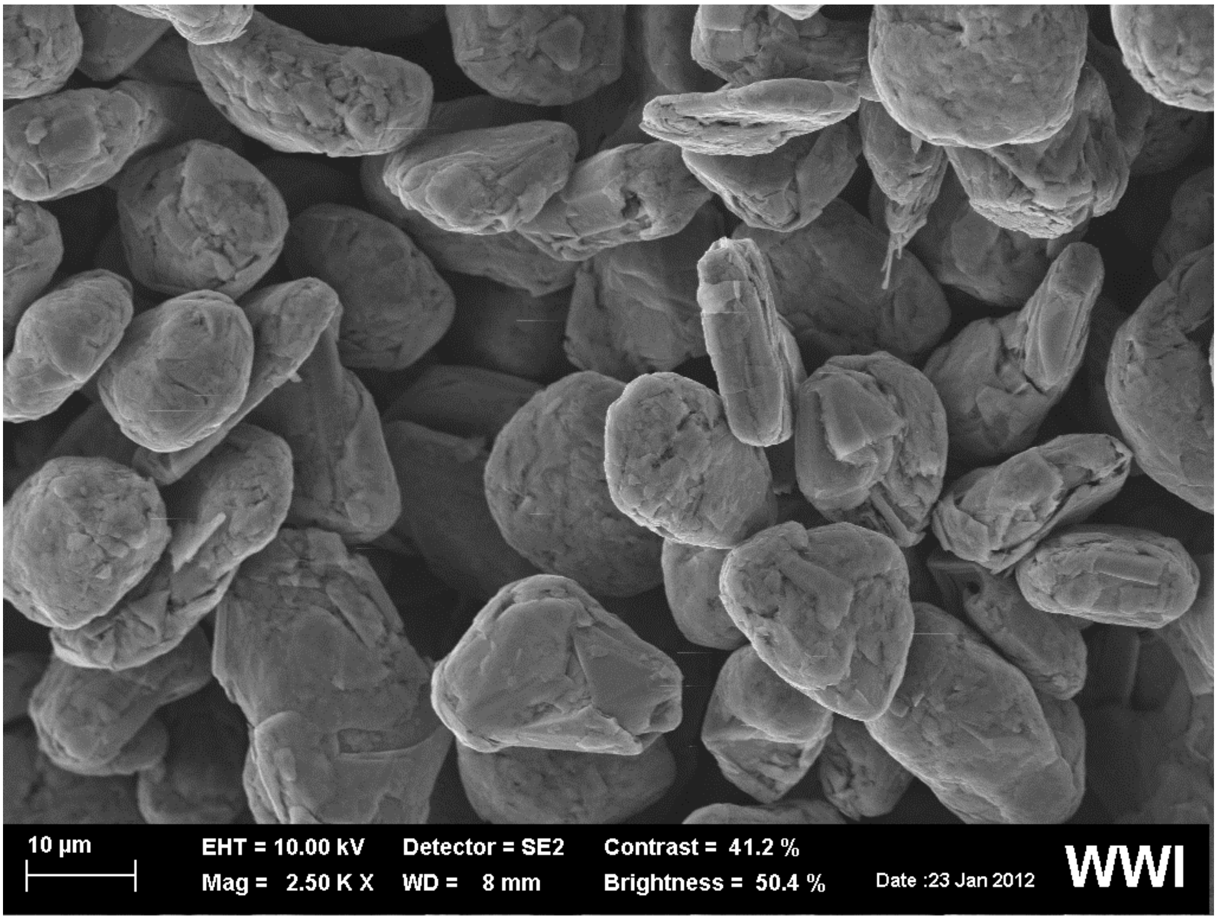

Supplementary Figure 5: SEM image of pristine material SGN18 graphite under 2,500x magnification.

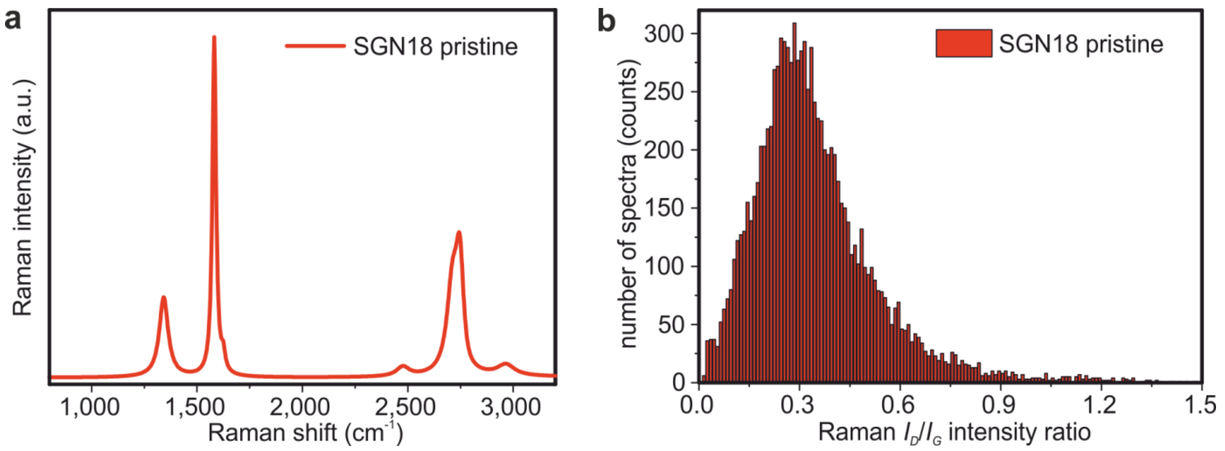

Supplementary Figure 6: **SRS analysis of the pristine graphite material SGN18 serving as reference G_P.** 10,000 spectra were recorded leading to a) the mean Raman spectrum and b) an I_D/I_G intensity distribution plotted as histogram.

Supplementary Table 1. Dilution series of benzonitrile and KC_n with n = 0 (solid K, Supplementary Figure 1), n = 8, 16, 24, 48 (Figure 1) for optical absorption measurements under inert conditions. The identical extinction at 390 nm was determined as ε_{390} = 4000 (± 50) L mol^{-1} cm^{-1}.

GIC	concentration of [K] in mol L^{-1} PhCN						
$K_{(s)}$	$5.1 \cdot 10^{-4}$	$3.4 \cdot 10^{-4}$	$2.6 \cdot 10^{-4}$	$2.1 \cdot 10^{-4}$	$1.6 \cdot 10^{-4}$	$1.4 \cdot 10^{-4}$	$1.2 \cdot 10^{-4}$
KC_8	$7.4 \cdot 10^{-4}$	$3.7 \cdot 10^{-4}$	$2.5 \cdot 10^{-4}$	$1.9 \cdot 10^{-4}$			
KC_{16}	$4.3 \cdot 10^{-4}$	$2.6 \cdot 10^{-4}$	$1.9 \cdot 10^{-4}$	$1.5 \cdot 10^{-4}$			
KC_{24}	$4.6 \cdot 10^{-4}$	$3.1 \cdot 10^{-4}$	$1.6 \cdot 10^{-4}$				
KC_{48}	$2.0 \cdot 10^{-4}$	$1.7 \cdot 10^{-4}$	$1.3 \cdot 10^{-4}$	$1.0 \cdot 10^{-4}$	$6.8 \cdot 10^{-4}$		

Supplementary Methods

Thermogravimetric Analysis combined with Mass Spectrometry (TG-MS). For the analysis of $^m/_z$ > 45 the thermogravimetric analysis of pure PhCN solutions was carried out on a Perkin Elmer Pyris 1 TGA instrument. Time-dependent temperature profiles in the range of 30 and 600 °C (20 K min^{-1} gradient) were carried out under a constant flow of N_2 (70 mL min^{-1}). About 2.0 mg initial sample mass was used. Online MS measurements were carried out with a GC-Clarus 680 with an Elite-5MS glass capillary column: 30 m length, 0.25

mm diameter. MS measurements of $^{m}/_{z} > 45$ (77, 103, 204) were performed on a MS Clarus SQ8C (Multiplier: 1800 V). The obtained data was processed with the TurboMass software.

For the analysis of $^{m}/_{z} < 45$ (H_2, H_2O) in the THF graphenide solutions in Figure 3, the TG-MS data was recorded on a Netzsch STA 409 CD instrument equipped with a Skimmer QMS 422 mass spectrometer (MS/EI) with the following programmed time-dependent temperature profile: 30-700 °C with 20 K min^{-1} gradient and cooling to 30° C. The initial sample weights were adjusted at 5.0 (±0.1) mg and the whole experiment was executed under inert gas atmosphere with a He gas flow of 80 mL/min. The obtained data was processed with the Netzsch Proteus Analysis software.

Supplementary References

1. Ralph W.G. Wyckoff. Crystal Structures Vol.1, Second edition, Interscience Publishers, New York, 7-83 (1963).
2. D. P. DiVincenzo and S. Rabii. Theoretical investigation of the electronic properties of potassium graphite. *Phys. Rev. B* **25**, 4110-4125, (1982).
